# Development of A Novel High Throughput Photo-catalyst Screening Procedure: UV-A Degradation of 17α-Ethinylestradiol with Doped TiO_2_-Based Photo-catalysts

**DOI:** 10.3390/ma13061365

**Published:** 2020-03-18

**Authors:** Tony B. Engelhardt, Sabine Schmitz-Stöwe, Thomas Schwarz, Klaus Stöwe

**Affiliations:** Institut für Chemie, Fakultät für Naturwissenschaften, Technische Universität Chemnitz, Straße der Nationen 62, 09111 Chemnitz, Germany; tony-benjamin.engelhardt@s2012.tu-chemnitz.de (T.B.E.); sabine.schmitz-stoewe@chemie.tu-chemnitz.de (S.S.-S.); thomas.schwarz@chemie.tu-chemnitz.de (T.S.)

**Keywords:** high throughput screening, photocatalysis, TiO_2_, endocrine disruptors, SPE, GC-MS

## Abstract

The rising pollution of surface water by endocrine disruptive chemicals (EDCS) could lead to the persistent harm of aquatic wildlife. Addressing this concern, advanced waste water treatment techniques should be established in addition to the present sewage treatment. Therefore, the promising advanced oxidation process of photocatalysis is discussed. With the aim of establishing a novel high throughput screening approach for photocatalysts, a workflow resting upon the use of a self-constructed 60-fold parallel stirring UV-A LED photoreactor, followed by parallel sample extraction by SPE and sequential automated analysis by GC-MS, was developed, and is presented in this article. With the described system, TiO_2_-based photocatalysts, doped with different amounts of zinc, and synthesised by a sol-gel-route, were tested regarding their activity in the photocatalytic degradation of the synthetic estrogen 17α-ethinylestradiol. Thereby, the functional behavior of the photoreactor system and its applicability in a high throughput process could be evaluated. As a result of the catalyst screening, TiO_2_ catalysts with low amounts of zinc were found with a significantly higher activity, compared to undoped TiO_2_. In conclusion, the presented system provides an easily accessible high throughput method for a variety of photocatalytic experiments.

## 1. Introduction

Considering the growing human population [1] and ever-expanding industries, humankind is confronted with an increasing quantity of pollutants in sewage and the landscape [2]. A wide variety of substances is discharged into nature [3,4], but in particular only one type of them aroused special attention in the past, the endocrine disruptive chemicals (EDCs) [5]. They are known for their ability to interfere with the hormone system of vertebrates, and influence it in a way restricting these species in their fertility and ability to mate with conspecifics [6,7,8]. While the database lags of information about the influence of EDCs on the human body, there are nevertheless indications that the negative effects of these chemicals observed in wildlife could also be carried over to humans as well [9,10]. Examples for this group of hazardous pollutants are steroids, naturally-formed, like estron (E1), 17β-estradiol (E2) and estriol (E3), or synthetically-obtained, such as 17α-ethinylestradiol (EE2), which is used in hormonal contraceptives. These estrogens are excreted by animals as well as by humans, hence finding their way into nature.

In the case of EE2, this coherence is especially worrisome, because this molecule was intentionally designed to be particularly stable against biological degradation, and to be highly estrogenically active [11]. To maintain the conditions of fresh water, municipal sewage treatment plants are introduced to the hydrological cycle, but it has been reported in literature that it is not possible to sustainably remove a plurality of micropollutants, and therefore EDC, from our wastewaters with the widespread activated sludge process [12,13,14,15]. In addition to this common type of sewage treatment, advanced oxidation processes (AOPs) like ozonation [16,17,18,19,20,21], or physical removal techniques like nanofiltration [22,23,24,25] and adsorption to charcoal [26,27,28,29,30], have been discussed lately. While these additional treatments deliver satisfying results regarding the reduction of micropollutants, they are inseparably linked to certain disadvantages, such as the generation of hazardous products and the disposal of adsorbents. Another, and presumably the most promising treatment procedure, is the photocatalysis [31,32,33,34], which is another variant of an AOP. Concerning the semiconductor materials utilised in this process, titanium dioxide (TiO_2_) is the most established and examined one [35,36,37,38,39,40,41,42,43]. Apart from the fact that it is already known that estrogens could be degraded satisfactorily with the help of photocatalysis making use of TiO_2_, only few information has been gathered respecting reaction kinetics, matrix effects, reaction products, doped TiO_2_ catalysts, and other semiconductors completely differing in their composition from TiO_2_ for the deployment in photocatalytic reactions. For targeting these research subjects in depth, it is essential to implement procedures which enable a convenient work flow for executing a vast number of experiments in a reasonable amount of time. Thus, this publication addresses the introduction of a high throughput photocatalysis procedure, resting upon the use of a UV-A light-emitting diode (LED) array in a multi-position stirring photoreactor, combined with parallel sample preparation by solid phase extraction (SPE), and the sequential automated analysis by gas chromatography coupled to mass spectrometry measurements (GC-MS) for the research on the degradation of EE2, as this technique provides qualitative as well as quantitative information about the catalyst sample. Furthermore, other high throughput compatible analysis designs were considered. On the one hand, a colorimetric analysis approach was prepared, utilising a reaction according to Kober [44,45,46,47,48], together with UV-Vis measurements with a microplate reader. Applying the Kober reaction to the colourless EE2 forms a pink-coloured molecule which principally allows its concentration determination in aqueous solutions, but the formation of by-products during photocatalysis made it difficult to specify the degradation of EE2 exactly. The by-products cannot be individually measured by this colorimetric method. On the other hand, the dynamic on-column reaction chromatography according to Trapp et al. [49,50,51,52] was discussed as a suitable technique, but its application has been postponed so far.

## 2. Results

### 2.1. Calibration

**Figure 1:**
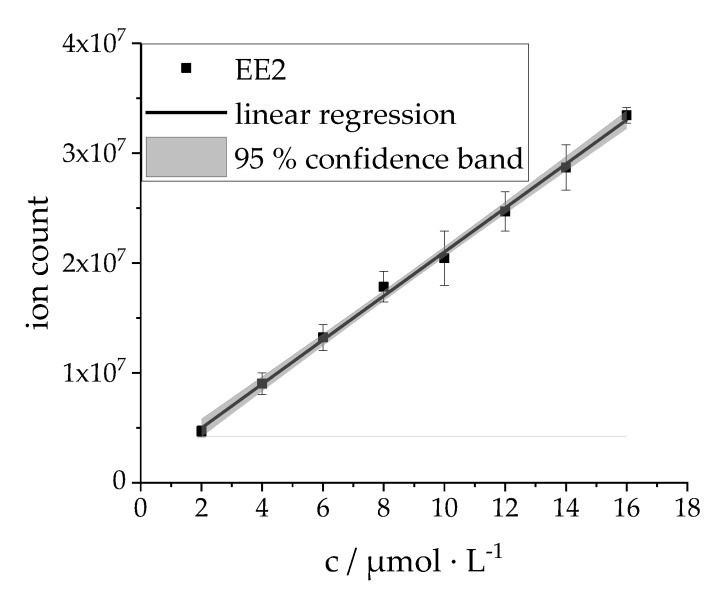
Calibration curve formed by gas chromatograph-mass spectrometry (GC-MS) measurements of eight calibration standards ranging from c = 2 µmol∙L^−1^ to c = 16 µmol∙L^−1^, with an increment of ∆c = 2 µmol∙L^−1^; quintuple determination; slope:2003070 L mol^−1^, point of intersection with the ordinate:971408.3; ion counts determined by the integration of the respective EE2 signal in the obtained EIC chromatograms (EIC = extracted-ion current; precursor ions: 440 m/z, 425 m/z, 285 m/z); the concentration values given are a representation of the EE2 concentration in aqueous solution during catalytic screening experiments.

Calibration measurements have been performed according to the workflow described in Section 4.2.3. The calibration data, as depicted in Figure 1, provides information about the process standard deviation (PSD), the limit of detection (LOD) and the limit of quantitation (LOQ). These values were calculated according to Deutsches Institut für Normung (DIN) 32645, and are given in Table 1.

As shown in Figure 1 and Table 1, the present calibration gives a satisfactory linearity for the whole range of measurements (R^2^ = 0.998), alongside a low LOQ. Concentration values above c = 16 µmol∙L^-1^ lead to saturation of the analytic system, and had to be left out. Regarding the execution of photocatalytic degradation experiments, a saturation of the system is ruled out, as the initial concentration of EE2 in aqueous solutions was set to c_EE2, initial_ = 15 µmol∙L^−1^. With the aim of catalyst screening (as described in Section 2.2: Photocatalytic experiments) a target concentration of 50 % (c_EE2, 50 %_ = 7.5 µmol L^−1^) in relation to the initial concentration should be reached during irradiation. This value resembles an optimal point for comparing different catalysts activities, as there is enough of the analyte degraded to show the photocatalytic degradation effect. At the same time the PSD has only a small bearing on the measured values (relative standard deviation = 3.3%).

The calibration experiments demonstrate that the presented GC-MS analysis is a suitable technique to quantify the photocatalytic degradation of EE2, especially in the discussed concentration range from c = 7.5 µmol∙L^−1^ to 15 µmol∙L^−1^.

### 2.2. Photocatalytic Experiments

#### 2.2.1. Catalyst Screening

**Figure 2:**
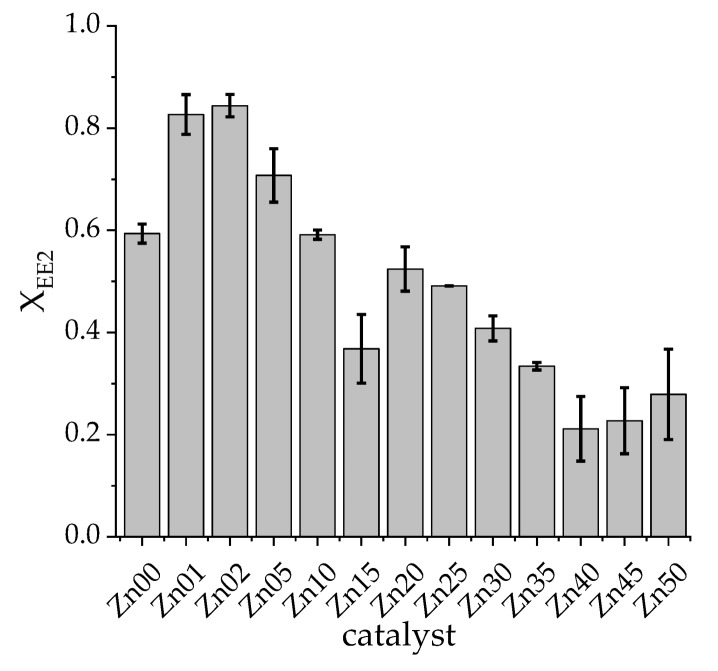
Catalyst screening of titanium dioxide-based photocatalysts, doped with different amounts of zinc synthesised by a sol-gel method; the number in the catalyst’s name (Zn25) indicates the amount of zinc in comparison to the amount of titanium used for synthesis in mol-percent; Zn00 resembles pure TiO_2_; conversion of EE2 X_EE2_ was studied; double determination; irradiation time = 3.5 min.

The conversion Χ_EE2_ of EE2 has been calculated for different photocatalysts, as defined in Equation (1). In Figure 2 the resulting conversions are shown. For the catalyst screening, defined EE2 amounts have been dissolved in deionate, and the solvent has been exchanged prior to GC-MS analyses by SPE from water to methanol followed by derivatisation of the analyte as described in detail in Section 4.2.5. Different amounts of zinc doping based on titanium dioxide were tested with the aim of finding a mixed oxide, which is more active regarding the degradation of EE2 than pure titanium dioxide.

The abbreviation ZnXX describes zinc-titanates, where XX indicates the amount of zinc in comparison to the amount of titanium in mol-percent. By performing the experiment leading to the data shown in Figure 2, the total capacity of the reactor system was not completely utilised, although all of the reactions, together with repetitions resulting in the error bars shown in Figure 2 had been carried out at the same time. It is shown that the addition of zinc to TiO_2_ is not promoting the photocatalytic activity of titanium dioxide in general. The EE2 conversion only benefits from zinc yields lower than 10% (compare Zn05, Zn02 and Zn01, in Figure 2).

(1) $${Χ}_{EE2}=1-\frac{n_{EE2,t}}{n_{EE2,\;0}}$$

#### 2.2.2. Reaction Monitoring

Another important part of photocatalysis research is the reaction monitoring. Collecting data from this kind of experiments makes important kinetic values, like the rate constant k, accessible, if the following is considered: During a heterogeneous catalytic reaction, the concentration of the catalyst remains constant throughout the photocatalytic reaction, which leads to the reaction rate r only being dependent upon the concentration change of the reactant molecule, in this case EE2. Therefore, the rate constant k can be calculated from the reaction rate r, as follows.

(2) $$r=-\frac{dc\left(EE2\right)}{dt}=k\cdot c\left(EE2\right)$$

(3) $$c_{t}\left(EE2\right)=c_{0}\left(EE2\right)\cdot e^{-kt}$$

(4) $$\frac{-\ln\left(\frac{c_{t}\left(EE2\right)}{c_{0}\left(EE2\right)}\right)}{t}=k$$

**Figure 3:**
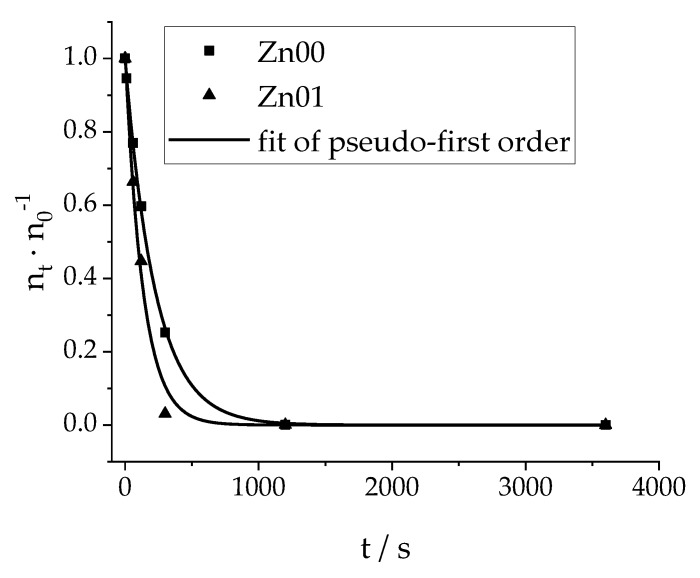
Reaction monitoring of the photocatalytic degradation of EE2 with porous titanium dioxide catalysts; the number in the catalyst’s name (Zn25) indicates the nominal amount of zinc in comparison to the amount of titanium used for synthesis in mol-percent; Zn00 resembles pure TiO_2_; each point resembles an individual formulation, n_t_ speaks of the ion count after the irradiation time t, n_0_ indicates the ion count without irradiation after dormant phase; the ion count is a measure of the concentration c (see the calibration curve in Figure 1); functional fit of pseudo-first order: k_Zn00_ = 0.0044 s^−1^, k_Zn01_ = 0.0075 s^−1^.

In Figure 3 the reaction monitoring of the photocatalytic degradation of EE2 in the presence of doped and undoped titanium dioxide after a dormant phase of t = 30 min without irradiation is shown. As seen there, a function of pseudo-first order describes the reactions kinetics perfectly, despite the fact that every data point collected resembles an individual reaction formulation (catalyst suspension in aqueous EE2 solution), and each data point of this reactions has been acquired with a different LED of the array (each reaction under another LED). Furthermore, it shows that the described reaction system provides a rapid degradation of EE2 with an almost quantitative conversion after 5 min (for zinc doped titanium dioxide, Zn01) and 20 min (for pure titanium dioxide, Zn00), this way, accomplishing significant faster complete depletion than other compatible systems [53,54,55,56]. E.g., Alcântara Marinho et al. [57] reported that under the best experimental conditions (pH = 8, c(Nano-TiO_2_; P25) = 250 mg∙L^−1^, UV-A radiation), and after 10 min, only 82% of EE2 was degraded.

### 2.3. Catalyst Characterisation

#### 2.3.1. Powder X-Ray Diffraction (PXRD)

**Figure 4:**
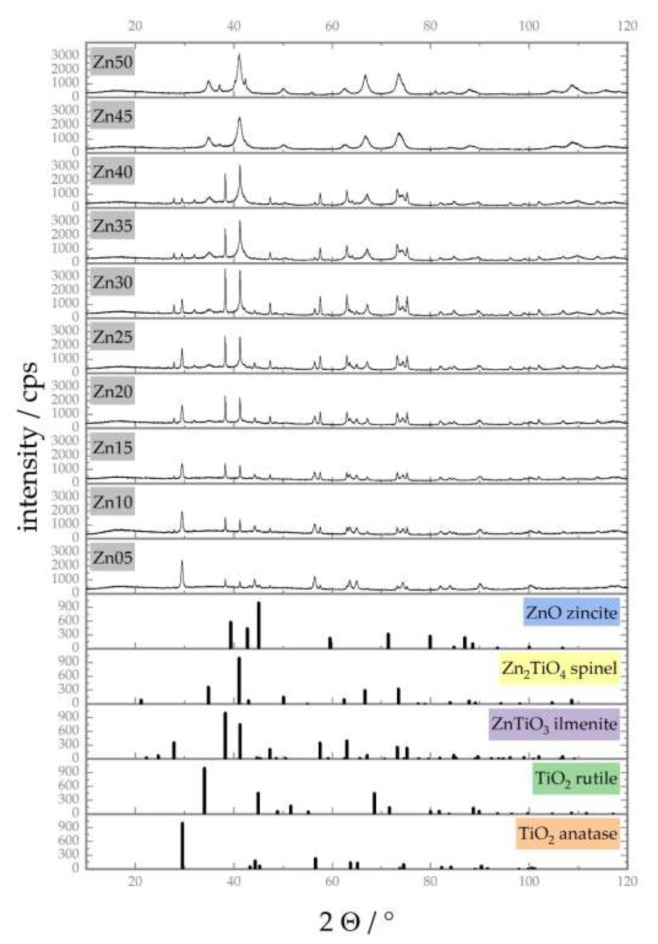
Powder X-Ray Diffraction (PXRD) pattern of catalyst materials Zn05 to Zn50, as well as reference data of TiO_2_ (anatase ICDD 73-1764 and rutile ICDD 73-1765), ZnTiO_3_ ilmenite (ICDD 85-0547), Zn_2_TiO_4_ spinel (ICDD 73-0578) and ZnO (ICDD 80-0075), acquired on a Bruker D8 diffractometer with Co fine focus tube (λ_Kα_ = 1.79021 Å, Θ-Θ geometry, VDS, Lynxeye detector). The number in the catalyst’s name (ZnXX) indicates the amount of zinc in comparison to the amount of titanium used for synthesis in mol-percent.

For the identification and quantitative determination of the crystalline phases characterising the catalytic materials synthesised, PXRD measurements have been performed. The measured diffraction patterns are given in Figure 4, including reference data for TiO_2_ in anatase and rutile modification, ZnTiO_3_ ilmenite, Zn_2_TiO_4_ spinel and ZnO for comparison and phase identification.

**Figure 5:**
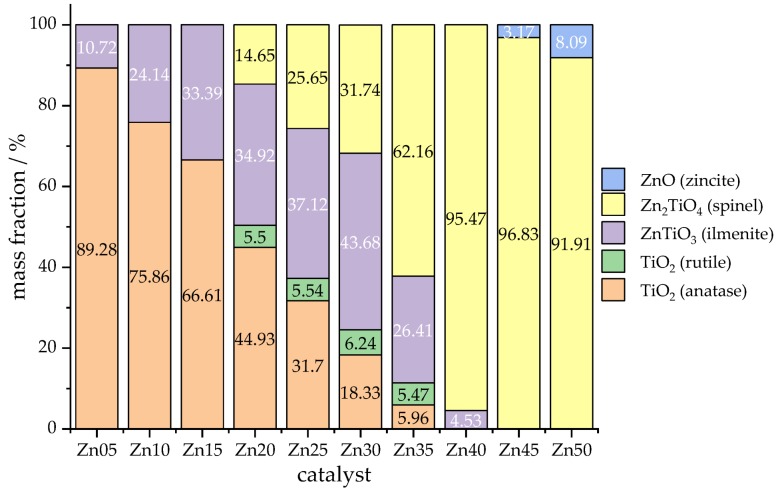
Relative crystalline phase compositions in wt.% of sol-gel-synthesised, zinc-doped titanium dioxide photocatalysts; determined by Rietveld refinement of the PXRD pattern; the number in the catalyst’s the (Zn25) indicates the nominal amount of zinc in comparison to the amount of titanium used for synthesis in mol-percent, lattice parameters of the anatase-type phases shown in Table 2.

The results of the quantitative phase analysis by Rietveld refinement of the diffraction pattern are shown in Figure 5. Depending on the nominal amount of zinc in the catalyst, different phase compositions occur. For lower amounts of zinc, only anatase and ilmenite phases were found (see Figure 5, Zn05, Zn10 and Zn15), while at higher zinc fractions, almost uniformly a zinc–titanium spinel was formed (see Figure 5, Zn40, Zn45 and Zn50). The appearance of a crystalline rutile phase to a minor amount (see Figure 5, Zn20, Zn25, Zn30 and Zn35) is not unexpected, as anatase is a metastable form of titania, transforming into rutile as the room temperature stable modification at a temperature of T = 600 °C [58]; thus, rutile might be formed by the phase transformation of anatase during calcination at atmospheric pressure (see Section 4.2.1 Catalyst Synthesis).

#### 2.3.2. Surface Determination by BET-Measurements

Based on the appearance of the adsorption and desorption isotherms measured by nitrogen adsorption experiments, the catalysts were received as nonporous materials with an average specific surface of 31.21 ± 2.40 m^2^ g^−1^.

## 3. Discussion

With the described methodology, it is possible to perform a variety of different photocatalytic experiments, such as catalyst screening, with the aim of gathering information about the activity and selectivity of several materials with distinct compositional doping and structural morphology. 

In the field of heterogeneous catalysis, it could be difficult to predict the effect of these parameters on the reaction, so it is common to test many different material compositions. Addressing this concern, the described 60-fold parallel stirring UV-A LED photoreactor system provides the possibility to carry out up to 60 photocatalytic reactions simultaneously, and this way resembles a powerful tool for the objective of photocatalyst screening.

According to the presented experiments, it has been shown that the applied photoreactor, combined with the automated sample processing by SPE, offers an effective possibility to test multiple (up to 60) titanium dioxide-based catalysts simultaneously in different photocatalytic experiments for their ability to degrade EE2 or other EDCs. With the help of a suitable calibration, it has been evaluated, that the analysis by GC-MS hits the requirements needed, as it provides a low LOQ alongside a tolerable PSD. The chromatographic step provided by GC-MS evokes a bottleneck in the complete high-throughput processing of EE2 degradation analysis, firstly because GC-MS has to be carried out sequentially, and secondly because a complex pretreatment procedure for the chromatographic processing is necessary, depending on the derivatisation of analyte molecules. This way it represents the time determining component for the whole process. The advantage of using a GC-MS analysis, is that it is not only feasible to gather information about the activity of a certain catalyst and the reaction rate, in addition it provides information about occurring metabolites. This way, a detailed insight in the degradation mechanism of the reactant molecule can be provided, which will be subject in a future publication. Furthermore, multiple analytes can be investigated in one sample, and this in a way that matrices of different molecules and their interference with each other during photocatalysis can be examined. These coherences should be discussed in a future publication, too.

Moreover, it was accomplished to emphasise that the activity of titanium dioxide benefits from the addition of low amounts of zinc (<10% according to the amount of titanium) to the mixed oxide regarding the degradation of EE2 in aqueous solutions. In comparison, the rate constant could almost be doubled.

The fact that only low amounts of Zn in TiO_2_ lead to an improved activity, can be explained by two facts. The photocatalytically-active anatase modification of TiO_2_ is a defect variant of a close dense packing of oxygen ions, in which one half of the octahedral voids are occupied by Ti^4+^ ions in an ordered fashion. The doping reaction of ZnO into the anatase crystal lattice may be illustrated by Equation (5) using Kröger–Vink notation:

(5) $$ZnO\left(TiO_{2}\right)\;⇌\;Zn_{Ti}^{\prime \prime }+V_{O}^{..}+O_{O}^{x}$$

As pure ZnO adopts a Wurzite type crystal structure, and is observed in a rock salt-type structure only at very high pressure, a preference of Zn^2+^ for occupation of tetrahedral voids may be concluded. On the other hand, the occupation of tetrahedral or interstitial voids in the anatase lattice is theoretically also possible, but would lead to severe intercationic repulsion, due to short interatomic distances. As Equation (5) shows, the substitution of Ti^4+^ by Zn^2+^ ions is accompanied by the incorporation of oxygen vacancies in the densely packed oxygen lattice, most probably facilitating the photocatalytic decomposition of endocrine disruptive chemicals. On the other side, increasing the amount of Zn doping is resulting after reaching the solubility limit in the additional formation of an ilmenite phase, which is much less photocatalytically active. The ilmentite structure is an ordered defect variant of a hexagonal close packing of oxygen ions with occupation of octahedral voids by Ti^4+^ and Zn^2+^, and no oxygen vacancies. So, the benefit of doping TiO_2_ with Zn is lost. This coherence is strongly correlating with the phase composition, as small amounts of ilmenite seem to promote the photocatalytic activity of titanium dioxide (anatase), while the formation of a zinc titanium spinel appears to inhibit its photocatalytic activity. These results will be targeted in future studies more deeply.

For future synthesis approaches, the goal will be to improve the established template-sol-gel method to obtain porous materials, as it was not possible with the exact described procedure. It will presumably be possible to promote the photocatalysts’ activity further by the introduction of porosity and the increase of the specific area.

Due to the flexibility of the whole system, it will be possible to carry out photocatalytic experiments on several target molecules in various solvents, and matrices with different catalysts in different concentrations.

Ultimately, the described multi-position photoreactor is an enrichment for photocatalysis fundamental research with the purpose of investigating pathways to improve sustainability in savage treatment.

## 4. Materials and Methods

### 4.1. Materials

#### 4.1.1. Chemicals

Two different EE2 stock solutions were prepared, one in methanol (GC-MS-grade) for calibration concerns (c_EE2_ = 67 µmol L^−1^; EE2 grade: ≥ 98%), and the other one in deionised water (Seral Seradest SD4000 Vario, κ < 0.5 µS∙cm^−1^; Veolia Water Technologies Deutschland GmbH, Celle, Germany) for catalysis experiments (c_EE2_ = 15 µmol L^−1^). For catalyst synthesis, titanium(IV) tetraisopropoxide (97+%, Alfa Aesar Thermo Fisher, Karlsruhe, Germany), zinc (II) acetylacetonate (for synthesis), acetylacetone (Carl Roth GmbH + Co. KG, Karlsruhe, Germany), P123 (Pluronic^®^, triblock-copolymer) and methanol (SupraSolv^®^) were used as precursors. During the analysis protocol, deionised water and methanol were utilised for SPE (see „Sample Pretreatment for Chromatography”) cartridge conditioning and methanol for subsequent analyte elution. For GC-MS analyses (see „Analyses”) preparation and derivatisation acetone (SupraSolv^®^), N,N-dimethylformamide (SupraSolv^®^), N-methyl-N-(trimethylsilyl)trifluoroacetamide (MSTFA) were deployed and chrysene-d_12_ (c = 0.1 g L^−1^, Supleco) was used as internal standard. As GC carrier gas helium (Alphagaz, 99.999%, AIR LIQUIDE Deutschland GmbH, Düsseldorf, Germany) was used. These chemicals were obtained from SIGMA-ALDRICH Chemie GmbH, Taufkirchen, Germany. All chemicals were used as received, and without further purification.

#### 4.1.2. 60-Fold Parallel Stirring UV-A LED Photoreactor

**Figure 6:**
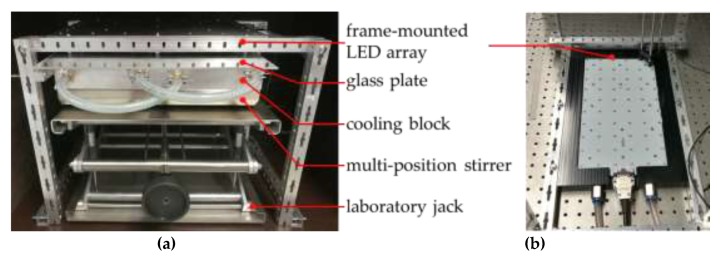
Applied photocatalysis reactor system (thermostats, controllers, light-tight box not shown): (**a**) Front view; (**b**) Bottom view of the UV-A light emitting diode (LED) array showing the 60 LED arrangement.

The entirety of photocatalytic reactions was realised with a self-constructed, 60-fold parallel stirring UV-A LED photoreactor, equipped with an LED array (λ = 365 nm, E = 384 mW cm^−2^, six rows of 10 LEDs; Cetoni GmbH, Korbussen, Germany), mounted in a frame self-built from aluminium square pipes, set by a controller (printed circuit board manufactured by CETONI, casing construction self-built) and temperature adjusted by a thermostat (Julabo F32; JULABO GmbH, Seelbach, Germany). The array was positioned above an aluminium cooling unit with inlaid cooling coils; the temperature of this unit was adjusted by a thermostat (Julabo F12; JULABO GmbH, Seelbach, Germany) and sample vial cavities (six rows of 10 cavities) for the reaction vials (screw cap bottles used without caps, clear glass, V = 8 mL, Ø = 16.6 mm; Lab Logistics Group GmbH, Meckenheim, Germany). Moreover, the aluminium block inhibited scattering radiation between the individual glass vials. During the radiation processing, the open-top vials were covered by a glass plate (fused silica FN08, thickness of 3 mm, Figure 6; GVB GmbH, Herzogenrath, Germany). For the purpose of homogenisation, this cooling unit was positioned on a multi-position stirrer (2mag MIXdrive 60; 2mag AG, München, Germany) set by another controller (2mag MIXcontrol 20 with RS232; 2mag AG, München, Germany). To adjust the distance between the LED array and the block holding the reaction vials, a laboratory jack (stainless steel, 400 × 400 mm, DIN 12897; Carl Friedrich Usbeck KG, Radevormwald, Germany) was introduced to the setup. The whole system (thermostats and controllers excluded) was operated inside a light-tight box, to prevent the emission of hazardous UV-A radiation during photocatalytic experiments, and to prevent light from the surroundings to interfere with the system. This described construction enables carrying out 60 photocatalytic reactions simultaneously, thus implementing a high throughput workflow.

### 4.2. Methods

#### 4.2.1. Catalyst Syntheses

Catalysts used during this study were obtained by following a sol-gel route, supported by using P123 as a template molecule. First, P123 was dissolved in methanol (7/3 (m/m)) and titanium-(IV) isopropoxide stabilised in acetylacetone (1/4 (n/n)). Next, zinc(II) acetylacetonate (mass depending on doping degree compared to the amount of titanium in the resulting catalysts) was dissolved in the P123/methanol solution, and afterwards the zinc(II) solution obtained was mixed with the titanium(IV) solution in the desired ratio. The resulting orange sol was then heated (T = 80 °C, t = 44 h) until a solid, (in the cases of too small an amount of stabiliser crystallised) gel was formed. Through the introduction of a calcination procedure (T_initial_ = 25 °C, ramp 1:2 °C∙min^−1^ to 250 °C, T_250 °C_ = 120 min, ramp 2:0.5 °C∙min^−1^ to 600 °C, T_600 °C_ = 480 min, natural cooling with closed furnace door) a colourless powder was acquired.

#### 4.2.2. Catalyst Characterisation

The synthesized catalysts were characterized by powder X-ray diffraction (PXRD, STOE StadiP, STOE & Cie GmbH, Darmstadt, Germany) and Brunauer–Emmett–Teller (BET, Quantachrome Instruments, Anton Paar QuantaTec Inc., Anton Paar GmbH, Graz, Austria) measurements. The PXRD measurements were performed on a Bruker D8 diffractometer with Co fine focus tube (λ_Kα_ = 1.79021 Å, Θ-Θ geometry, VDS, Lynxeye detector, Bruker Corporation, Billerica, USA) and refined by a Rietveld method with the program TOPAS 5.0; the BET surface determination with a NOVAtouch 4LX (adsorbate: nitrogen; pore size, according to Dollimore-Heal).

#### 4.2.3. Calibration

The EE2 stock solution for calibration has been prepared in methanol with a concentration of c_EE2_ = 67 µmol∙L^−1^ (see Section 4.1.1 Chemicals). Eight calibration standards of EE2 ranging from c = 2 µmol∙L^−1^ to c = 16 µmol∙L^−1^, with an increment of ∆c = 2 µmol L^−1^ have been prepared by diluting the appropriate volume of stock solution in a 100 mL volumetric flask, with methanol at constant temperature of 25 °C. All calibration standards have been stored in a dark surrounding before measurement to avoid any degradation by light. Each calibration standard has been measured five times (quintuple determination) using the analysis workflow described in Section 4.2.6. As the calibration standards neither include water nor catalyst, it was not necessary to undergo a pretreatment for chromatography (see Section 4.2.5).

#### 4.2.4. Photocatalytic Reaction

A standard procedure was introduced for the purpose of catalyst screening described in the following. The prepared catalyst powders were weighed out (m = 7.5 mg/vial) directly into the reaction vials, and the desired estrogen solution (V = 5 mL, c = 15 µmol L^−1^) was added, before they were inserted into the cavities of the photoreactor. The cooling unit containing the vials were adjusted to its closest position to the LED array (space between light source and the solutions surface 3.08 cm). The catalyst libraries were subsequently stirred without irradiation (f_rot_ = 1000 min^−1^, dormant time t = 30 min, T = 20 °C) for equilibration purposes, before the irradiation tests were started.

#### 4.2.5. Sample Pretreatment for Chromatography

**Figure 7:**
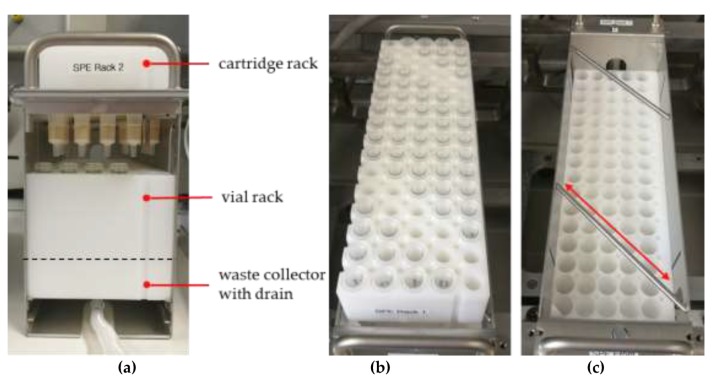
Solid phase extraction (SPE) rack: (**a**) front view (rack removed from the chem station) holding cartridges and vials; (**b**) top view, cartridge rack, implemented into the chem station; (**c**) top view, vial rack, implemented into the chem station.

**Figure 8:**
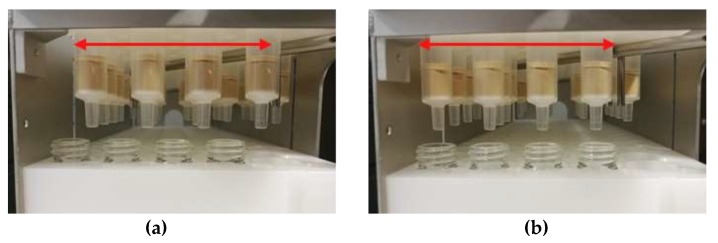
Representation of the SPE rack’s functionality: (**a**) Position A: conditioning and sample introduction; (**b**): Position B: elution.

Before the GC-MS measurement, a sample preparation had to be performed using SPE followed by derivatisation. The application of a chem-station (Chemspeed, Accelerator SLT 106) made it possible to execute the SPE semi-automatically. For this purpose a tripartite SPE rack (Chemspeed) was used, equipped with up to 80 SPE cartridges (Agilent Bond Elut – PPL, m_solid phase_ = 200 mg, V_cartridge_ = 3 mL) in its topmost part (referred to as cartridge rack, Figure 7a), and accordingly up to 80 vials (LLG Labware, screw cap bottle, clear glass, V = 8 mL, Ø = 16.6 mm) in the part below (referred to as a vial rack, Figure 7a), for the purpose of collecting the eluents. The cartridge rack is relocatable to enable the adjustment of two different positions (Figure 7b,c). One is described as waste position (Figure 8a, position A). In this position, conditioning solvents and residue solution after sample introduction flowing through the cartridges are bypassing the vial rack through drill holes inside of it, and directly reach the SPE-racks bottommost part, the waste collector (Figure 7a). This waste collector is fitted with a drain and directly connected to a waste container. Adjusted to the other position (Figure 8b, position B), described as elution position, eluents containing the analytes were collected by the corresponding vials. The SPE procedure started with the automated conditioning of the cartridges with pure solvents (1. methanol, V = 3 mL, Q = 3 mL∙min^−1^; 2. deionised water, V = 3 mL, Q = 3 mL∙min^−1^, rack position A), followed by the manual application of the sample suspensions (rack position A). Afterwards, the analytes were eluted from the cartridges by solvents dispensed by the 4-needle head (4NH) of the chem station in an automated fashion (methanol, V = 3 mL, Q = 3 ml∙min^−1^, position B, recovery rate = 100%), and collected as described. The following steps were carried out manually after transferring the samples vials to a sample rack accommodating up to 80 samples. The methanolic solutions obtained were dried by evaporation (T = 80 °C) on a precision hot plate (Harry Gestigkeit GmbH, PZ 72) and the residues again dissolved in acetone (V = 1.5 mL), with the purpose of transferring them into GC vials (Macherey-Nagel, vial N9, flat bottom, threaded, labelled). To each of the GC vials a solution containing the internal standard (V = 90 µL) was added. Afterwards, the solvent was evaporated again (T = 65 °C). The dried analytes were then derivatised (T = 65 °C, t = 30 min) with MSTFA (V = 100 µL) in N,N-dimethylformamide (V = 100 µL).

#### 4.2.6. Analyses

Analyses were carried out with a GC-MS (Agilent GC 7890B, Column: Agilent DB-5, Ø = 0.25 mm, film thickness = 0.25 µm, length = 30 m; Agilent MS 5977B MSD with quadrupole mass analyser). Samples were conveyed fully automated by a multipurpose auto sampler (Gerstel MPS robotic pro), handling a micro syringe (Gerstel 65 mm USM, V = 10 µL), deriving the liquid samples from GC-vials, and injecting them directly into a programmed temperature vaporising (PTV) injector (Gerstel CIS 4 (CIS = cold injection system), liner: Gerstel liner for CIS 4, baffled, deactivated). The injector was connected to an active cooling device (Gerstel UPC plus (UPC = universal politer cooling)). Initialising the measurement, the MPS injected the sample (V = 2 µL) directly into the injector’s liner (T_init_ = 130 °C, t_init_ = 0.5 min), in which the solvent volume was reduced by using the Solvent-Vent-Mode. For this reason, the split-valve was kept open (t = 0.3 min) to evaporate the solvent, and was closed afterwards, before the injector was heated (12 °C s^−1^ to 300 °C, t_300°C_ = 10 min). The sample evaporated completely, and was transferred into the GC’s separation column, where the GC oven started the pre-set heating program (T_init_ = 120 °C, T_120°C_ = 1 min; ramp 1: 20 °C min^-1^ to 230 °C, T_230°C_ = 2 min; ramp 2: 5 °C min^−1^ to 280 °C, T_280°C_ = 1 min) alongside the set carrier gas flow (helium, Q = 1 mL∙min^−1^, p = 80.3 kPa). After the analytes passed the separation column, they reached the MS across the transfer line (T = 280 °C), which started its operation after a solvent delay (6 min) by ionising (EI = ion impact ionisation, T_EI source_ = 230 °C) the components. The ion fragments generated this way were then filtered according to their mass-to-charge ratio by a quadrupole analyser (T_Quad_ = 150 °C, (m/z)_min_ = 50, (m/z)_max_ = 700, resolution = 0.1 m/z, scan speed = 781 u/s, scan frequency = 1.2 scans/s). The total ion current was detected.

#### 4.2.7. High Throughput Workflow

**Figure 9:**
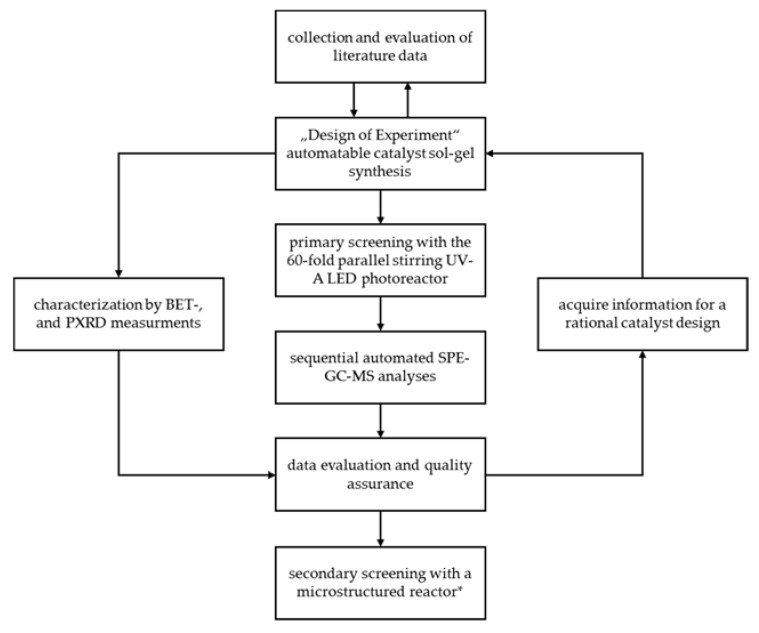
High throughput workflow for photocatalyst research utilising the 60-fold parallel stirring UV-A LED photoreactor during primary screening; *in progress.

With the gathered information a high throughput workflow was designed as shown in Figure 9.

## 5. Conclusions

A high-throughput workflow for photocatalyst research has been elaborated, which includes the synthesis and characterisation of catalyst materials, a photocatalytic degradation process of EE2 using a self-constructed 60-fold parallel stirring UV-A LED photoreactor, and a sequential automated SPE-GC-MS-analysis process for quantitative and qualitative determinations. This workflow cycle has been carried out for a primary screening of Zn-doped titanium oxides. It could have been shown that it is possible to gain an almost quantitative EE2 degradation after t = 5 min of irradiation (λ = 365 nm, E = 384 mW∙cm^−2^) by using 1 mol % zinc-doped titanium dioxide (Zn01). This corresponds to a four-fold increase of the reaction rate compared to undoped titanium dioxide (Zn00).

In order to improve the photocatalytic properties of the investigated materials further, the developed workflow has to be repeated analogously with other doping elements and element combinations. A future challenge will be speeding up the most time-consuming process step in this high-throughput workflow—the SPE-GC-MS analysis.

## Figures and Tables

**Table 1:** Values for process standard deviation (PSD), limit of detection (LOD) and limit of quantitation (LOQ); referring to Figure 1; according to DIN 32645.

PSD/µmol L^−1^	LOD/ppm	LOQ/ppm
0.25	0.56	1.09

**Table 2:** Lattice parameters refined by Rietveld method for the crystalline anatase-type phase according to the PXRD measurement results depicted in Figure 5.

Catalyst	Lattice Parameters/Å	
	a	c
Zn05	3.7840(1)	9.5063(3)
Zn10	3.7851(1)	9.5043(4)
Zn15	3.7851(1)	9.5033(4)
Zn20	3.7853(1)	9.5004(4)
Zn25	3.7849(1)	9.5036(3)
Zn30	3.7850(1)	9.5026(4)
Zn35	3.7853(3)	9.4928(1)

## Abbreviations

The following abbreviations are used in this manuscript:

AOP	advanced oxidation processes
BET	Brunauer–Emmett–Teller
c	concentration
CIS	Hcold injection system
DIN	Deutsches Institut für Normung
E	irradiance
EDC	endocrine disruptive chemicals
EI	ion impact ionisation
EIC	extracted-ion current
E1	estron
E2	17β-estradiol
E3	estriol
EE2	17α-ethinylestradiol
f_rot_	rotation speed
GC-MS	gas chromatography coupled to mass spectrometry
init.	initial
κ	conductivity
k	rate constant
λ	wavelength
LED	light-emitting diode
LOD	limit of detection
LOQ	limit of quantitation
m	mass
MSTFA	N-methyl-N-(trimethylsilyl)trifluoroacetamide
MPS	multi-purpose auto sampler
m/z	mass-to-charge ratio
mol%	mole fraction
n	amount of substance
n_t_	ion count after the irradiation time t
n_0_	ion count without irradiation
P123	Pluronic®, triblock-copolymer
PSD	process standard deviation
PTV	programmed temperature vaporising
PXRD	powder X-ray diffraction
Q	flow
R^2^	coefficient of determination
SPE	solid phase extraction
T	temperature
t	time
UPC	universal politer cooling
UV-A	ultra violet A
V	volume
v	reaction rate
wt%	mass fraction
X_EE2_	conversion of EE2
Zn XX	Zinc-titanate; XX indicates the amount of zinc in comparison to the amount of titanium in mol-percent
